# Steroids for the Prevention of Sensorineural Hearing Loss Secondary to Acute Otitis Media: A Systematic Review

**DOI:** 10.1055/s-0045-1811640

**Published:** 2026-04-24

**Authors:** André Filipi Brandão Santos Sampaio, Rafael da Costa Monsanto, José Jarjura Jorge, Godofredo Campos Borges

**Affiliations:** 1 Service d'Oto-Rhino-Laryngologie et Chirurgie Cervico-Faciale, Centre Hospitalier Universitaire d'Angers, Angers, Pays de la Loire, France; 2 Department of Otorhinolaryngology, Pontifícia Universidade Católica de São Paulo, Sorocaba, SP, Brazil; 3 Department of Otolaryngology – Head & Neck Surgery, University of Minnesota Twin Cities, Milwaukee, MN, United States; 4 Department of Otorhinolaryngology – Head and Neck Surgery, Escola Paulista de Medicina, Universidade Federal de São Paulo, São Paulo, SP, Brazil

**Keywords:** otitis media, sensorineural hearing loss, inner ear, steroids

## Abstract

**Introduction:**

Acute otitis media (AOM) is associated with the development of permanent sensorineural hearing loss (SNHL). The potential role of steroids in preventing cochlear damage secondary to AOM has been discussed.

**Objective:**

To critically analyze the current evidence on the use of steroids to prevent AOM-associated SNHL.

**Data synthesis:**

A total of 15 studies was categorized into 3 groups: 1) studies on histopathological changes in the inner ear secondary to AOM (n 5); 2) those on the relationship between AOM and hearing outcomes (n = 5); and 3) studies on the (hearing or histological) outcomes of AOM treatment using steroids (n = 5). Experimental studies in animals and human temporal bones revealed that AOM is associated with the upregulation of proinflammatory cytokines in the middle and inner ears, resulting in an inflammatory process. Inflammatory cytokines and bacterial toxins translocate from the middle to the inner ears through the semipermeable round window membrane, causing structural damage to the neuroepithelium, mainly in the cochlear basal turn. In experimental studies, the use of steroids has been shown to reduce the expression of inflammatory cells and cytokines and the structural damage affecting the stria vascularis and hair cells. One study clinically evaluated the effects of steroids on AOM patients with SNHL, and it demonstrated significant improvements in hearing thresholds.

**Conclusion:**

Experimental data demonstrate that steroids can reduce the expression of inflammatory cytokines and reduce structural damage to the cochlear neurosensory epithelium. However, these findings have yet to be translated to a clinical setting due to the lack of high-level evidence.

## Introduction

Acute otitis media (AOM) is a disease with high incidence and prevalence worldwide, especially in the pediatric population, being the top-ranked condition and leading patients and caregivers to seek pediatric emergency departments. It can affect people at any age, but the peak incidence ranges from 6 to 24 months of age. 1 The incidence in adults is much lower compared with children. Studies 2 3 have described the incidence of AOM in adults as ranging from 2.7 to 5.3 per 1 thousand person-years.

In general, the full spectrum of clinical presentations of otitis media (OM) may cause complications associated with substantial morbidity and mortality, 4 but it can also cause varying degrees of hearing sequelae, including the development of permanent sensorineural hearing loss (SNHL); 5 6 OM may also cause secondary endolymphatic hydrops (SEH), leading to more severe degrees of hearing loss and varying degrees of vestibular symptoms. 7 Although SNHL is more frequent in cases of chronic suppurative OM, 8 recent studies 5 have revealed that even a single AOM episode can result in permanent high-frequency (HF) hearing loss. A cohort study 9 10 of 30 years demonstrated that patients with a childhood history of chronic suppurative OM or recurrent AOM had worse hearing, as well as higher prevalence of self-reported tinnitus and vestibular symptoms, as compared with peers without a history of those diseases, suggesting a role of continuous inflammation in the development of these symptoms. The true incidence of SNHL in AOM patients is not clear.

The diagnosis of AOM is based on the characteristic clinical symptoms and otoscopic findings. Most cases can be treated with pain control and careful observation, while patients with bilateral disease and/or severe symptoms (toxemia, fever > 39 °C, otalgia for > 48 hours) are treated with antibiotics. 1 11 The role of steroids in AOM management is still unclear: currently, their use is restricted to selected cases or in research protocols. Recent evidence 12 13 indicates that steroids might be useful in the prevention of cochlear damage secondary to OM, either by reducing local cytokine production or by reducing direct damage to inner-ear structures. Additionally, steroids have been associated with lower rates of treatment failure and shorter duration of middle ear effusion, potentially reducing the risk of OM sequelae such as SNHL. 13 14 15 16

Considering the potential benefits of steroids in preventing AOM-related SNHL, we conducted a systematic review of the literature to critically analyze the current evidence regarding their use in preventing SNHL secondary to AOM.

## Review of Literature

### Search Strategy

From September 2021 to April 2023, we conducted a systematic review of the literature in the he PubMed, LILACS, SciELO, and Scopus databases using the following search strings: *sensorineural hearing loss acute otitis media* and *acute otitis media steroid* , with a restriction to articles published within the last 20 years. Additionally, we screened the reference lists of the selected articles and gray literature (Google Scholar) to identify any further relevant studies.

### Study Selection

All studies identified in the preliminary selection (title and abstract screening) were assessed in full by two of the authors (AFBSS and RdCM). In cases of disagreement, a third independent reviewer (JJJJ or GCB) made the final decision on whether the study should be included in the final analysis. We included original studies reporting the hearing outcomes of AOM patients. The exclusion criteria were studies whose abstract or full text was unavailable; studies written in languages other than English, Portuguese, and Spanish; and studies including populations with genetic diseases or syndromes, chronic OM, or a history of ear surgery. Some of the parameters collected were the pathogen involved in AOM, the number and species of subjects, the duration of the intervention/treatment, and the methods of outcome assessment.

Reference screening and backward citation tracking were performed to identify any missed articles. Full-text screening and data extraction were conducted by two authors (AFBSS and RdCM). Assessment of the quality of the experimental studies using validated scales was not possible.

## Results

### Study Selection

**Fig. 1:**
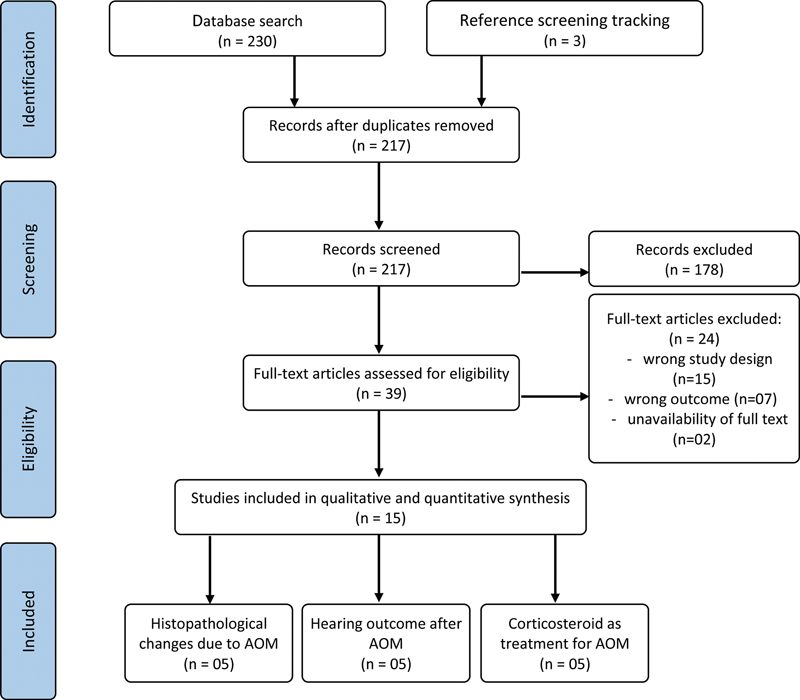
Flowchart of the criteria for the inclusion of studies in the systematic review.

The initial search yielded 230 articles; an additional 3 were identified through reference tracking ( Fig. 1 ). After removing duplicates, 217 studies remained for title and abstract screening, resulting in the exclusion of 178 (83,2%) articles. Following full-text review, 24 articles were excluded due to study design (n = 15), irrelevant outcomes (n = 7), or unavailability of the full text (n = 2). Ultimately, 15 studies 5 7 12 13 16 17 18 19 20 21 23 24 25 26 27 met all inclusion criteria and were selected for appraisal.

Due to the significant heterogeneity in study designs and non-standardized reporting of results, the included articles were categorized into 3 groups: 1) studies on histopathological changes in the inner ear secondary to AOM 17 18 19 20 21 (n = 5); 2) studies investigating the relationship between AOM and hearing outcomes 5 7 23 24 25 (n = 5); and 3) studies evaluating the effects (either hearing or histological outcomes) of the steroid treatment for AOM 12 13 16 26 27 (n = 5).

In the first group, 17 18 19 20 21 animals' models were used to evaluate the response of the middle and inner ears to AOM. The analysis period following inoculation with AOM pathogens ranged from 24 hours to 28 days. These studies 17 18 19 20 focused on evaluating the expression of inflammatory mediators and cytokines in the middle and inner ears using various laboratory techniques. One study 21 also assessed auditory brainstem response (ABR) threshold shifts in an AOM animal model, comparing the results to those of wild-type control mice.

Group 2 5 7 23 24 25 focused on evaluating the hearing sequelae of AOM, and it included 220 subjects. Three of the studies used a healthy control group for comparison; 23 24 25 one used the contralateral ear (in cases of unilateral AOM) as a control, 5 and one did not include a control group in their analysis. 7 The articles in group 3 12 13 16 26 27 (evaluating the role of steroids in AOM treatment) included a total of 153 rats 12 13 16 27 (experimental studies) and 7 humans 26 (a clinical study), who received steroids at varying doses. The most commonly used steroid was dexamethasone, a glucocorticoid.

Quality assessment of the six cohort studies 5 7 23 24 25 26 involving patients was conducted using the Newcastle-Ottawa Scale. 22 Four of the studies 5 23 24 25 was classified as “high-quality”, while the remaining two 7 26 was classified as “low-quality”.

### Histopathological Changes Due to AOM

Different animal models of AOM were studied, including mice, guinea pigs, and chinchillas. To induce AOM, both *Haemophilus influenzae* and *Streptococcus pneumoniae* were used ( Table 1 ).

**Table 1:** Studies on histopathological changes in acute otitis media

Authors	Bacterium	Animal	Method/Period	Findings
** MacArthur et al., 17 2011 **	*Streptococcus pneumoniae*	BALB/c mice (n = 18)	qRT-PCR 24 hours after inoculation	Significant elevation of middle-ear mRNA for the inflammatory cytokines IL-1 , IL-1ß , IL-6, TNFα, and BMP7, with IL-6 showing a near 8-fold change. Cochlear tissues showed increased expression of these same cytokines, as well as of FGF1, TGFb, and VEGFa. The strongest response was seen in IL-1b, with more than 3- fold. BMP7 and IL-6 appeared to be expressed at significantly lower levels than normal.
			Immunohistochemistry after 24 hours	IL-1 and IL-1β stained in a similar pattern, seen in the middle-ear epithelium, tympanic membrane, and inner-ear lateral wall (including both the stria vascular and underlying spiral ligament). Staining was also seen in the organ of Corti and spiral ganglion. IL-6 stain was noted in the middle-ear epithelium and tympanic membrane. Also, TNF was observed in the inner-ear lateral wall
** Ghaheri et al., 18 2007 **	*Haemophilus Influenzae*	BALB/c mice (n = 20)	Cytokine gene expression at 3 and 7 days	The major upregulated cytokines in acute disease were the macrophage M-CSF, FGF, interleukins (ILs), interferons (IFNs), tumor necrosis factor (TNF) and tumor necrosis factor-stimulating factors (TNFSF), BMP, and vascular endothelial growth factors (VEGFs), all of which showed increasing expression with prolongation of inflammation from 3 to 7 days. Some anti-inflamatory genes and cytokines were downregulated.
			Histology at 3 and 7 days	The entire middle-ear space was often filled with fluid and inflammatory cells, demonstrating the severe reaction to the bacterial products. By 7 days, most of this inflammation had subsided.
			Immunohistochemistry at 3 and 7 days	NF-kB was commonly found in the inner ear. Cochlear staining also was seen for FGF and BMP. Inflammatory factors such as IFN-a and CSF were also seen in the middle and inner ears.
** Trune et al., 19 2015 **	*H. Influenzae*	BALB/c mice (nws)	RT-PCR analyses at 6, 24, and 72 hours, and at 1 week	Both middle and inner ears showed a similar pattern between gene expression and its product. IL-1, IL-1β, IL- 6, MIP-1, MIP-2, G-CSF, and KC were upregulated. Generally, the magnitude of the changes was comparable between the middle and inner ears, both peaking early and declining rapidly after that, with many proteins having their highest level measured at 24 hours.
			Quantitative ELISA At 18, 24, 36 and 48 hours	
			Immunohistochemistry after 1 day	Staining was observed at the middle and inner ears, especially for IL-6 and TLR-4. Other cytokines involved were IL-1, MIP-2, G-CSF and TLR 2 and 9. The specific location of staining was different for each cytokine; however, the spiral ligament, spiral ganglion, lateral wall, and stria vascular appeared brighter.
** Watanabe et al., 20 2001 **	Bacterial lipopolysaccharide	Guinea pigs (n = 12)	Immunohistochemistry at 48 hours	Myeloperoxidase immunoreactivity was evident in the structures of the spiral ligament and the stria vascular, as well as in the supporting cells of the organ of Corti, Hensen's cell and Deiter's cell, but not in the sensory cells.
			Electrocochleographic recording after 48 hours	The threshold shift of the compound action potential (CAP) was elevated significantly after 48 hours in the LPS group.
** Tsuprun et al., 21 2008 **	*S.pneumonia* ; wild- type (40 CFU/mL) (4 animals); PspA-deficient mutant (5 animals); Ply-deficient mutant (5 animals); and PBS (5 animals)	Chinchillas (n = 19)	Auditory brainstem response at 28 days	The animals inoculated with the wild-type showed ABR threshold losses of 10 to 15 dB for 4 to 32 kHz and more than 20 dB for 1 to 2 kHz. Therte were statistically significant differences across all frequencies of ABR thresholds.
			Histopathology at 28 days	No fluid, inflammatory cells or bacteria were observed in the middle ears. Only the animals inoculated with wild-type *S. pneumoniae* showed structural changes: the inner ear showed slight strial edema and marginal cell bulging and protrusions in the stria vascularis in 1 animal and translucency of the intermediate cells in 2 animals. No sign of labyrinthitis or any missing outer or inner hair cells were observed in the sections of any cochlear turn. Other cochlear structures, including spiral ganglion cells, seemed to be intact.
	Higher-dose *S. pneumoniae* (∼ 10 ^3^ CFU) (3 animals)	Chinchillas (n = 3)	Histopathology at 5 days	1 animal had severe edema of the intermediate cells of the stria vascularis and 1 had severe damage of the stria.

**Abbreviations** : ABR, auditory brainstem response; BMP, bone morphogenetic proteins; CFU, colony-forming units; FGF, fibroblast growth factors; G-CSF, ranulocyte colony stimulating factor; IL, interleukin; KC, keratinocyte-derived chemokine; M-CSF, macrophage colony stimulating factor; MIP, macrophage inflammatory protein; qRT-PCR, quantitative real-time polymerase chain reaction; TGF, transforming growth factorv; TLR, toll-like receptor; TNF, tumor necrosis factor; VEGF, vascular endothelial growth factor.

MacArthur et al. 17 suggested that inflammatory interleukins (ILs) were produced locally in the cochlea of animals with AOM. It has been shown that some areas of the cochlea, such as the spiral ligament (SL) and the lateral wall, contain fibrocytes that play a role in the production of cytokines upon stimulation by bacterial components through receptors of the *toll-like receptor* ( *TLR* ) *2* ( *TLR2* ), *4* ( *TLR4* ), and *9* ( *TLR9* ) genes. 17 Ghaheri et al. 18 and Trune et al. 19 demonstrated that tissues within the cochlea, particularly the SL of the lateral wall, can express cytokine messenger RNA (mRNA), leading to local cytokine production. The increase in local cytokine levels initiates and amplifies the intracochlear immune response. 18 19

Sensorineural hearing loss secondary to AOM can also be related to structural cell damage caused by free radicals. The presence of an inflammatory process may disrupt the homeostasis of the myeloperoxidase-catalyzed system, which is responsible for supplying energy to the inner ear, leading to the accumulation of reactive oxygen species and subsequent degeneration of cellular structures within the scala media. In the organ of Corti, it appears that the degeneration of supporting cells precedes damage to the cochlear sensory cells. 20

Tsuprun et al., 21 in animals treated with wild-type *S. pneumoniae* and the pneumococcal surface protein A (PspA) and pneumolysin (Ply) strains, observed mild hearing loss and slight structural changes in the stria vascularis (SV), suggesting that the pneumococcal PspA and Ply proteins may contribute to the damage to the SV that results in SNHL. Additionally, moderate SNHL has been reported after the application of IL-1β and IL-8 onto the round window membrane (RWM) of rats, with no morphologic changes observed in the cochlea. 21

### Hearing Loss Secondary to AOM

In the studies in group 2, 5 7 23 24 25 comprising a total of 220 patients, the subjects were followed-up for different periods of time ( Table 2 ), and all studies used at least standard tonal audiometry to measure the outcomes.

**Table 2:** Studies on acute otitis media (AOM) and hearing outcomes

Authors	Number of patients	Treatment/Follow-up	Audiologic tests				
			Standard audiometry	Impedance audiometry	Extended high-frequency audiometry	Vocal audiometry	Transient click-evoked otoacoustic emissions
** Cordeiro et al., 5 2018 **	41 patients (41 ears)	Antibiotics only/0; 14, 28, 49 and 180 days after	First appointment: presence of air- bone gap and conductive hearing loss in all ears affected by AOM. At 14 days, standard audiometry did not show hearing loss in the ears affected by AOM.	The tympanometry performed at the last evaluation demonstrated type-A curves for all 82 ears.	The extended high-frequency (8–16 kHz) audiometry demonstrated significant threshold elevations in the affected ears, which were consistently higher in the affected ears in the subsequent follow-up Appointments.	No significant differences in the speech recognition thresholds and discrimination scores between the affected and the contralateral ears at the last follow-up appointment.	Not available.
** Park et al., 7 2014 **	75 patients (83 ears)	Oral antibiotics, otic antibiotic-steroid dops, and oral steroids, if required/not specified (range: 0.6–46.3 months)	8 out of 83 ears (75 patients) showed sensorineural hearing loss during AOM course (> 30 dB).	Not available.	Not available.	The mean speech reception threshold (SRT) was of 51.8 dB, and the speech discrimination score was of 100% in all patients.	Not available.
** Kasemodel et al., 23 2020 **	27 patients (30 ears) with AOM and 16 controls (32 ears)	Not specified/7 days	Among patients with AOM, the auditory thresholds were significantly worse compared to the control group ( *p* < 0.001). The bone thresholds of AOM patients were also significantly worse than those of the controls at all frequencies.	20 out of 30 ears with AOM (66.67%) showed a type-B curve, whereas 5 (16.67%) showed a type-C curve. One ear showed a type-A curve, and in 4 ears it was not possible to perform the immittance testing due to the presence of tympanic.	Not available.	Worse speech recognition thresholds (mean of 38.5 dBHL in the AOM group versus 7.65 dBHL in the control group).	Not available.
** Ryding et al., 24 2002 **	33 patients: 13 children with recurrent AOM (rAOM) and 21 controls	Not specified/follow-up investigation when they had reached the age of 10 years	There was no significant difference between the groups regarding any of the hearing level thresholds for the frequencies of 125–8 kHz, and there were no air- bone gaps.	On tympanometry, the maximum compliance on both sides was higher in the rAOM children. The middle-ear pressure was significantly lower in the rAOM children than in the controls. Reflexes could not be evoked at any frequency on either side in 3 of the 12 rAOM children on contralateral stimulation. In contrast, reflexes could be evoked in all 21 controls.	Children with a history of recurrent AOM exhibited significantly higher hearing level thresholds at most frequencies measured as compared with the controls; those with the higher number of AOM episodes generally had higher hearing threshold levels than those with a lower number of AOM episodes.	The mean speech discrimination scores were worse in children with a history of rAOM as compared with the controls.	The right-ear response was significantly weaker in the rAOM group as compared to the controls in the frequency bands of 4 and 5 kHz. Similarly, the left-ear response was weaker in the frequency bands of 1, 4, and 5 kHz in the rAOM group ( *p* < 0.05)
** Krakau et al., 25 2017 **	28 participants (13 with rAOM and 15 controls)	Not specified/30 years latter	A difference between the groups was found at 1,000 and 3,000 Hz, air thresholds, on right ears. For all other comparisons, there were no differences.	Impedance audiometry reported normal values for all subjects in both groups.	Elevated hearing level thresholds in the rAOM group as compared to the controls for high-frequency (HF) audiometry. When evaluated separately, the statistical results for the right and left ears, respectively, showed a significant difference (p = 0.05) at 10 kHz for the right ear and at 9, 10, and 12.5 kHz for the left ear.	Not available.	Not available.

Using conventional tonal and speech audiometry, Kasemodel et al, 23 showed increased bone- and air-conduction thresholds among patients with AOM as compared to healthy patients, providing evidence of sensorineural hearing damage. Similarly, Park et al. 7 showed persistent SNHL secondary to AOM in 9.3% of the ears affected by AOM, and some of the patients with AOM-associated hearing loss later developed tinnitus. By also analyzing HF audiometry, Cordeiro et al. 5 further demonstrated that the extended HF hearing thresholds were still higher as compared with controls after 6 months of AOM.

Regarding long-term sequelae, Ryding et al. 24 and Krakau et al., 25 who also performed standard and HF audiometry, demonstrated that the HF thresholds of patients with a history of AOM were significantly higher than those of non-affected patients after 10 and 30 years respectively. Transient click-evoked otoacoustic emissions were performed by Ryding et al., 24 who identified that the wave thresholds in both ears were significantly weaker in the recurrent AOM group as compared to the controls in the frequency bands of 4 and 5 kHz.

None of these articles identified the pathogen involved in AOM-associated SNHL.

### Prevention of AOM-related SNHL or Ear Damage Using Steroids

The studies evaluating the role of steroids in the prevention of inner-ear damage and hearing loss included experimental protocols 12 13 16 27 (153 mice/rats) and a clinical evaluation 26 (7 humans) ( Table 3 ). In the experimental studies, the three most common pathogens ( *S. pneumoniae* , *Moraxella catarrhalis* , and *H. influenzae* ) were used to induce AOM. The selected studies did not standardize the type, dose, or administration method for the steroid treatment, and the results were evaluated through audiologic tests, histopathological methods, and cochlear blood flow (CBF) analysis.

**Table 3:** Studies on the steroid treatment for acute otitis media (AOM)

Authors	Number of patients	How AOM was induced	Steroid	Time of the treatment	Results	
					Methods	Inflammation measures
** Sone et al., 12 2003 **	16 Female Sprague- Dawley rats divided into 4 groups: LPS; steroids; NO; PBS.	*Escherichia coli*	30 μl of 1% dexamethasone dissolved in PBS inoculated intratympanically.	The steroids were provided 30 minutes after middle-ear inoculation with LPS and were examined 24 hours after.	Histopathological analysis of the middle ear	All the middle-ear cavities of rats that received LPS were filled with fluid, but the volume of fluid was lower in the group treated with steroids or ONO-1714.
					Cochlear blood flow (CBF)	The CBF ratio was of 0.8, 0.95, and 1.09 in rats that received PBS, dexamethasone, and ONO-1714 respectively, with a significant difference among them. The average values of the changes in normalized CBF 30 minutes after application of prostaglandin E1 were of 1.06, 1.11 and 1.18 respectively. The increase in rats treated with PBS or dexamethasone was significantly lower than in the control rats.
					Electron microscopic findings	The stria vascular showed slight enlargement of intercellular spaces and projection of the marginal cells towards the endolymphatic space. In comparison with the control rats or the ones who received PBS, there was a better aspect, but rats treated with ONO-1714 following LPS showed the stria vascularis of almost.
** Jang et al., 27 2007 **	10 Male Sprague- Dawley rats divided into 2 groups: one that received LPS and another that received steroids and PBS.	*Pseudomonas aerugionosa*	Dexamethasone 40 μL intratympanically	1 hour after LPS inoculation, the rats received treatment and were examined 24 hours after.	CBF	Intratympanic dexamethasone inoculation to the round window of rats led to a statistically significant increase in CBF compared to the PBS-treated group. The recovery rate of vascular conductance compared to the left side was significantly higher in the topical dexamethasone-treated group (0.92) than in the PBS-treated group (0.75). The response to round window application of PGE1 in the dexamethasone-treated group was better than in the PBS-treated group.
					Auditory brainstem response threshold	The intratympanic LPS inoculation showed ABR threshold elevations which were larger at higher frequencies. The elevated threshold recovered significantly after intratympanic dexamethasone administration, compared to the PBS-treated group.
** Park and Yeo, 16 2001 **	27 Male Sprague- Dawley rats divided into 3 groups: untreated, treated with penicillin, and treated with and penicillin and dexamethasone.	*Streptococcus pneumoniae*	Penicilin associated with dexamethasone, which was administered intramuscularly (1 mg/kg)	Initiated 2 days after bacterial inoculation and continued for 5 days. On days 4, 7, and 14 after inoculation, 3 randomly selected animals from each group were examined	Middle-ear findings	Inflammatory response was least severe in the group treated with steroids. The healing of the tympanocentesis site was the slowest. In the group treated with antibiotics and steroids, the changes were least pronounced on days 4 and 7. On day 14, the mucosal findings resembled those of the untreated controls, meaning that infiltration of inflammatory cells, submucosal thickening, and vascular dilatation had gradually subsided, whereas the metaplasia from squamous epithelium to ciliated epithelium persisted,
** MacArthur et al., 13 2009 **	100 BALB/c mice divided into 5 groups: control and the 4 types of steroids. Both ears were inoculated and examined.	*S. pneumoniae*	Oral prednisolone (5 mg/kg/day)	One day before inoculation and continued until they were killed (3 or 5 days)	Histopathological analysis	In comparison, glucocorticoid-treated ears showed a significantly higher incidence of inflammation-free ears. The prednisolone-treated mice had 10 MEs without symptoms, while 11 dexamethasone-treated ears were clear (statistically significantly better than the controls). Mineralocorticoid-treated ears were no different than those of the controls ( *p* > 0.05).
			Dexamethasone SC (0.75 mg/kg/day)		Cell number	The number of inflammatory cells was significantly reduced in all steroid groups compared with the controls. Prednisolone and dexamethasone appeared to reduce cell number, but the post-hoc test results showed statistical significance only between the prednisolone and fludrocortisone results.
			Oral fludrocortisone (10 μg/kg/day)		TM thickness	The glucocorticoid-treated groups were significantly different from the controls for TM thickness at day 3. The reduction in TM thickness measured in the mineralocorticoid-treated mice was not different from that of the controls, despite a lower mean value. The TM thickness showed an overall group effect for the steroids, and they did not differ from each other at day 5.
			Oral aldosterone (15 μg/kg/day); one ear from a 5-day aldosterone-treated mouse was not available for evaluation, leaving 19 ears in this group		Overall	None of the steroid results were different from each other; however, for the dexamethasone-treated mice, the mean fluid area was statistically significantly smaller compared with the control mice. There was an overall group effect for the steroids, and they did not differ from each other on day 5. However, the dexamethasone-treated mice were statistically significantly different from the controls. Thus, the results of the steroid treatments were generally similar in reducing ME inflammation during the observation period. Post-hoc comparisons generally showed that one or both glucocorticoids were better at reducing inflammation than the mineralocorticoids. However, all steroids were effective in reducing the number of inflammatory cells by day 5, suggesting that some.
** Heywood et al., 26 2016 **	7 humans with unilateral SNHL.	Not identified.	Initially, 7-day course of oral antibiotics and oral prednisolone. Due to failure of improvement, methylprednisolone was offered as 3 weekly intratympanic injections (0.5 mL of 40 mg/mL), followed by a 1-week course of dexamethasone 0.1% drops applied 4 times a day after the first infection	The first intratympanic injection was given at a mean of 29 days after the onset of symptoms (range, 13-42 days)	Pure tone audiometry	One out of 7 had improvement in PTA of 52 dB. Three out of 7 who on presentation showed SHNL isolated to the high frequencies (4 and 8kHz) achieved a return to normal hearing across all frequencies (mean improvement of 15 dB pre- and post-ITS). Among the non-responders, two presented with mostly severe sensorineural loss across and progressed to a profound loss across all frequencies, despite treatment. The other one had a mild-to-moderate low frequency loss, which remained unchanged despite treatment.

**Abbreviations** : LPS, lipopolysaccharide; ME, middle ear; NO, nitric oxide; PBS, phosphate-buffered saline; PGE, prostaglandin E; SNHL, sensorineural hearing loss; TM, tympanic membane.

Using electron microscopy, Sone et al. 12 found minimal abnormalities in the groups treated with dexamethasone or nitric oxide 30 minutes after middle-ear inoculation of endotoxin, while animals treated with phosphate-buffered saline showed further signs of stria vascularis degeneration (enlargement of intercellular spaces in the intermediate cells).

Jang et al. 27 compared intratympanic saline solution and intratympanic dexamethasone in AOM-induced animals. Tests performed 24 hours after AOM induction showed complete recovery of the ABR audiometry threshold in the steroid-treated group, as well as an increase in cochlear blood flow in comparison to the controls.

After inducing unilateral AOM, Park and Yeo 16 divided 27 rats into 3 groups: 1) rats that were not treated; 2) those that received intramuscular antibiotics; and 3) animals that received both intramuscular antibiotics and steroids (daily for 5 days). Although the antibiotic-treated group showed a beneficial effect on the mucosal changes, more pronounced and obvious effects on the inflammatory response were observed in the group that received both drugs, including the preventive effect of mucosal metaplasia to secretory epithelium which are related to an increased risk of recurrent AOM and OM with effusion.

MacArthur et al. 13 compared the effects of mineralocorticoids (improvement in fluid homeostasis) and glucocorticoids (causing immune suppression) in the inner ear response. Both treatments were generally similar in reducing middle- and inner-ear inflammation. Post-hoc comparisons showed that both classes of steroids were effective in reducing the number of inflammatory cells by day 5.

Heywood et al., 26 who the only clinical study, included patients with unilateral AOM who developed SNHL despite a 7-day course of oral antibiotics. After placement of a Sheppard ventilation tube, topical steroids were administrated (3 intratympanic injections of methylprednisolone 40 mg/mL followed by dexamethasone drops at 0.1% 4 times a day for 1 week). Four out of 7 patients improved their thresholds either in their pure-tone average or in the HFs (4 and 8kHz). The variables associated with a prognosis of good hearing recovery were patients who had mild SNHL affecting exclusively the HFs and patients who received antibiotics within the acute phase of AOM. Nevertheless, patients presenting with more severe hearing loss affecting all audiometric frequencies and those who presented with persistent hearing loss after AOM resolution had a worse prognosis for hearing recovery.

## Discussion

One of the many complications that can arise from AOM, SNHL results in the highest long-term burden. In a pilot cost-of-illness study on the long-term complications/sequelae of AOM, 28 the group that presented permanent SNHL had the highest mean cost. Unfortunately, no clinical treatment has been proven effective in the prevention or treatment of AOM-associated SNHL; therefore, treatments that could prevent these sequelae are highly warranted. There is a lack of high-quality evidence to support the prevention of AOM-related SNHL. Clinical practice guidelines 29 30 and Cochrane systematic reviews 31 do not endorse the use of steroids for the treatment of AOM. However, these guidelines and reviews have evaluated the use of steroids for AOM resolution without considering the risk of SNHL in the equation.

Inflammatory mediators and bacterial toxins can translocate to the inner ear through RWM, resulting in intracochlear production of ILs and inflammation. The lateral wall structures are important for ion homeostasis; inflammation can affect potassium (K + ) transport and its recycling into endolymph, resulting in hearing loss. Inflammation can also affect the tight junctions of endothelial cell that are necessary for preservation of the blood-labyrinth barrier. 19 Paparella et al. 32 and Ferster et al. 33 have also reported that OM can result in SEH from a variety of mechanisms, including pressure variations in the middle ear, RWM mobility alterations, or by the resulting intracochlear inflammatory process itself. The RWM seems to be the main route for the passage of inflammatory mediators and products to the inner ear; 34 however, other routes might also be involved in this process (such as the oval window and middle-ear vessels). 35 It is not possible to predict exactly when the damage to the inner ear begins, but inflammatory aspects were already observed 24 hours after inoculation by immunohistochemistry. 17 19

Acute otitis media leads to rapid cytokine production in the middle and inner ears – including IL-1α, IL-1β, and IL-6, tumor necrosis factor, and bone morphogenetic protein – that begins within 24 hours, peaks around 72 hours, and tends to decline thereafter. 17 18 19 During the peak of inflammation, cytokines involved in inflammatory response and tissue remodeling are upregulated. 18 These studies demonstrated that cytokines are locally produced by structures within the lateral wall and the organ of Corti, 17 18 19 20 and are not solely the result of passive translocation through the RWM. 36 Furthermore, studies 20 21 have revealed significant threshold shifts 48 hours and 28 days after bacterial inoculation, which were not attributable to conductive hearing loss, demonstrating transient and permanent SNHL secondary to AOM.

We hypothesize that, although the production of proinflammatory cytokines may reduce the risk of labyrinthitis and propagation of the infection to the central nervous system, it could, in turn, increase the chances of inflammatory lesions to the cochlear sensory epithelium.

Five clinical studies have focused on evaluating short- and long-term hearing sequelae secondary to AOM. Hearing loss in patients with AOM during the acute phase of the disease has been observed, 23 and although it could be argued that this threshold shift might be temporary and secondary to the active inflammatory process, robust evidence suggests otherwise. Permanent hearing loss affecting the extended HFs has also been observed 6 months after AOM resolution, 5 similar to a case report published by Margolis and Nelson. 37 High-frequency thresholds were significantly elevated among patients with a history of recurrent OM, long after the acute disease. 24 25 Collectively, these results clearly demonstrate that the inner ear suffers not only early functional damage (most likely inflammatory) during the AOM episode but also permanent structural damage. Although the clinical significance of such extended HF hearing loss is still not fully understood, studies 38 39 suggest that it is associated with tinnitus, deterioration of musical tone perception, and difficulties in sound localization and speech perception, potentially resulting in academic underachievement.

Hydén et al. 40 studied patients (n = 20) with AOM presenting with inner-ear complications and/or facial palsy. Since their focus was on the infectious agent, their study was not included in the present review, but the presence of SNHL in this scenario is noteworthy. Among the 15 patients with hearing loss, 5 had severe loss across the entire frequency range, 6 had loss in the mid and high frequencies, and 4 had HF loss.

Only 5 studies 12 13 16 26 27 have evaluated the use of steroids to treat or prevent SNHL secondary to AOM, including one clinical 26 and four experimental studies. 12 13 16 27 The only clinical study 26 advocating for the use of steroids has several shortcomings: the sample was very small, preventing a precise statistical analysis. Furthermore, the steroid regimen did not appear to follow the current recommendations from similar protocols, and there was significant heterogeneity in age and in the time elapsed between the onset of AOM symptoms and steroid treatment. Therefore, the results must be interpreted with caution. The experimental studies 13 27 41 unanimously corroborated the effectiveness of steroids in reducing middle- and inner-ear inflammatory cytokines, inflammation, and cochlear structural damage. Considering that steroids can effectively reduce the expression of inflammatory genes, they appear to be promising candidates to reduce AOM-related SNHL in a clinical setting, provided their safety and cost-effectiveness are confirmed. Moreover, structures within the scala media (SV, SL, and inner/outer hair cells) and spiral ganglion neurons possess steroid receptors. 41

Based on the data we have gathered, steroids appear to be a promising therapy for the prevention of AOM-associated SNHL. Several pathophysiological mechanisms identified in these studies can be adequately reversed or mitigated by steroid treatment, resulting in improved hearing outcomes and a lower risk of cochlear structural damage. Although steroids can theoretically be used in such cases, three critical questions remain unanswered by the current evidence. First: which patients are at risk of developing inner-ear sequelae secondary to AOM? Second: are the risks associated with steroid use outweighed by their potential benefits in these cases? Third: if indicated, how soon should steroid treatment be administrated? These are unresolved questions that should be further explored through experimental studies, as well as randomized, controlled, double-blinded clinical trials.

The small number of studies dedicated to evaluating SNHL secondary to AOM and steroid treatment or prevention, along with the high methodological heterogeneity among them, did not enable us to perform a meta-analysis of the outcomes. We could not restrict our research specifically to the pediatric population, as there are not enough studies; however, there is no reason to believe that these results could not apply to this group. Therefore, there is insufficient evidence to either support or dismiss the use of steroids in this specific population. Despite these limitations, our results suggest that steroids represent a promising therapeutic strategy to prevent SNHL being secondary to AOM. Studies on this topic are critically needed to evaluate their efficacy and safety before they can be translated into clinical practice. Since the pediatric population was not evaluated in any of the included studies, we cannot formally advocate for the use of steroids in children; however, the risk-benefit ratio should be carefully considered.

## Conclusion

Acute otitis media can lead to inner-ear damage through different pathophysiological pathways, potentially resulting in SNHL or elevated HF thresholds, which may be associated with tinnitus or difficulties in speech perception. Further exploration clinical and experimental studies are warranted before the use of steroids for AOM-associated SNHL can be translated into the clinical practice.
